# Correlates of exclusive breastfeeding practices in rural and urban Niger: a community-based cross-sectional study

**DOI:** 10.1186/s13006-019-0226-9

**Published:** 2019-07-30

**Authors:** Mami Hitachi, Sumihisa Honda, Satoshi Kaneko, Yasuhiko Kamiya

**Affiliations:** 1 Leading Program Graduate School of Biomedical Science, Nagasaki University, Nagasaki, Japan; 2 Department of Nursing Graduate School of Biomedical Sciences, Nagasaki University, Nagasaki, Japan; 3 Department of Eco-epidemiology Institute of Tropical Medicine, Nagasaki University, Nagasaki, Japan; 4 School of Tropical Medicine & Global Health, Nagasaki University, Nagasaki, Japan

**Keywords:** Exclusive breastfeeding, Optimal breastfeeding, Health professional, Traditional birth attendant

## Abstract

**Background:**

Exclusive breastfeeding (EBF) can prevent death and disease among young children. The proportion of EBF is low in Niger. This study aimed to identify the prevalence and correlates of exclusive breastfeeding.

**Methods:**

We conducted a community-based cross-sectional study in urban and rural areas of Niger among mothers of infants under 7 months old. We used a structured questionnaire to investigate breastfeeding practices, sociodemographic factors, and health service use. We used multivariate analysis to explore the correlates of EBF since birth.

**Results:**

The study involved 234 urban and 283 rural mothers. Colostrum was almost universally given to newborns (98.7% [231/234] urban and 97.9% [277/283] rural) and many mothers started breastfeeding within an hour of giving birth (69.2% [162/234] and 90.5% [256/283]). The proportion of EBF since birth in urban and rural areas was 15.8% (37/234) and 54.4% (154/283), respectively. Among mothers who had ceased EBF, proportion of prelacteal feeding was 85.3% (168/197) in urban areas and 62.0% (80/129) in rural areas, while 93.4% (183/196) and 72.7% (88/121) had stopped EBF within 1 week after birth respectively. The median duration of EBF was 1 week in urban and 2 months in rural areas. In urban areas, EBF was more likely in mothers with infants 3 months old or younger (Adjusted Odds Ratio [AOR] 2.78; 95% Confidence Interval 95% [CI] 1.07, 7.21) and problems with delivery including Caesarean section (AOR 3.60; 95% CI 1.17, 11.01). In rural areas, lower socioeconomic status (AOR 1.89; 95% CI 1.12, 3.18), early initiation of breastfeeding (AOR 4.04; 95% CI 1.50, 10.83) and delivery assisted by a traditional birth attendant (AOR 3.49; 95% CI 1.37, 8.89) were correlated with exclusive breastfeeding.

**Conclusions:**

Exclusive breastfeeding was uncommon. Most mothers ceased EBF within 1 week after birth. Adequate information about EBF by health professionals around delivery seems to encourage its use. To encourage EBF in Niger, it is important to educate health professionals, including traditional birth attendants, and enable them to discuss the practice with mothers through individual counselling or group education.

**Electronic supplementary material:**

The online version of this article (10.1186/s13006-019-0226-9) contains supplementary material, which is available to authorized users.

## Background

Exclusive breastfeeding (EBF) for 6 months after birth has been predicted to prevent 823,000 deaths in children under 5 years of age annually, by decreasing the risks of infectious disease and malnutrition [1]. Yet, the prevalence of EBF for 6 months remains low around the world, only 40% according to UNICEF figures [2]. Many studies on factors encouraging EBF have been carried out. Factors affecting breastfeeding practice are generally categorized into cultural, sociodemographic (e.g. urban or rural residence, level of education, parity), socioeconomic, healthcare related (e.g. the number of antenatal care visits, birth place), or related to physical condition of mothers and infants (e.g. birthweight, maternal and infant anthropometric status) and mother’s psychological condition (e.g. mother’s self-efficacy) [3–16]. It is therefore important to understand breastfeeding practice in the local context because these factors will vary.

The estimated population of Niger in 2012 was 16,274,738, and more than 80% lived in rural areas, where the lifestyle is quite different from urban areas [17]. Niger is one of the 15 countries with high child mortality, with its rate being 85/1,000 live births, and pneumonia and diarrhea occupy about one fourth of all causes of deaths [18, 19]. Furthermore, over 40% of infants were suffering from stunting [2]. More than half of the population had no access to clean water [2]. Exclusive breastfeeding is therefore an effective way to save children’s lives in Niger, and a number of national and international programs have already been implemented [20, 21]. Policies, strategies and action plans have been developed at the national level around three key breastfeeding practices: introduction of breastfeeding in the first hour after birth, EBF until 6 months after birth and continued breastfeeding with complementary foods until at least 24 months of age [22]. In addition, Niger has been implementing many of the provisions of the International Code for the Marketing of Breast Milk Substitutes and interventions like Baby-Friendly Hospital of UNICEF in collaboration with the United Nations and non-governmental organizations (NGO) [21]. Prenatal and postnatal care is conducted at the health center (Centre de Santé Intèglé) and health post (Cases de Santé) to support breastfeeding practices. However, the prevalence of EBF until 6 months of age in Niger was reported to be 23.3% with its median duration being 1.1 months in 2012 [23]. This prevalence was much lower than the national goal of 50% and that in some neighboring countries like Mali, Burkina Faso and Liberia (37.3, 50.1 and 54.6% respectively). It is therefore important to revise strategies to fit local contexts and use targeted promotion activities.

This study aimed to identify the prevalence and correlates of EBF and find out about feeding practice for infants under 7 months old in Niger.

## Methods

This study was conducted in one urban area (Niamey) and in one rural area (BirniN’Konni district) between July and October 2012. The study sites were selected from wards in urban areas and villages in rural areas. They are registered administratively as the smallest local units. Niamey, the capital of Niger, had an estimated population of 1,388,682 in 2012 with its mortality rate in children under 5 years being 80 per 1,000 birth lives [17, 23]. As first procedure of sampling, two of 44 health centers were chosen [17]. From eight wards covered by those two centers, two of three wards with health posts and three of five wards without health posts were selected as our study site. The rural area for this study was BirniN’Konni, which is one of the district of Tahoua region, about 417 km east of Niamey with its estimated population being 520,622 as of 2012 [17]. This region has a high child mortality rate; 140 per 1,000 live births [23]. Two of 17 health centers in this district were chosen considering accessibility to health services. Among 63 villages covered by those two centers, two of 15 villages with health posts and two of 48 without health posts were chosen randomly.

To be eligible and impartial for variables for breastfeeding, the participants of this community-based cross-sectional study were the biological mothers of infants under 7 months old living in the study sites. Mothers who had any breastfeeding difficulty because of medical conditions were excluded from this study. We recruited participants who lived along streets selected randomly in study site and were at home during our household visits. The participants were informed about our study and their rights, in the local language. Written informed consent was obtained prior to data collection. The child’s month of age was confirmed by mother and child health card or recall using the local event calendars, which had been made for this study.

We used a structured questionnaire with closed-ended questions to collect data about breastfeeding practices for infants, sociodemographic conditions and use of health services (Additional file 1). The trained local data collectors made interview with questionnaires translated into the Zarma or Hausa language. The dietary history in infants was assessed by mother’s recall regarding items which were selected from indicators recommended by WHO to assess feeding practices and local specific liquids and semi-solids [24]. In this study, breastfeeding practice was classified by the following criteria according to WHO and UNICEF; a) early initiation of breastfeeding, meaning initiation of breastfeeding within first hour after birth; b) exclusive breastfeeding (EBF), meaning the infant has only received breast milk, oral rehydration solution (ORS), vitamins, minerals and medicines since birth; c) predominant breastfeeding, meaning the infant who has received breast milk and certain liquids like water and fruit juice; d) complementary feeding, meaning the infant has received breast milk and solid or semi-solid foods and non-human milk and formula. The median duration of EBF was calculated based on infant’s month of age at the time of interview for EBF respondents and cessation for non-EBF respondents. The feeding practice in the 24 h prior to the interview was also asked but was not used for analysis of EBF.

We also asked about the type of house, well and primary water source and family assets to give a total score for socioeconomic status, categorized into higher or lower. The cut-off point depended on the median for each location, because there were significant differences confirmed using the Wilcoxon rank sum test. The score of nine or more was used for a higher socioeconomic status in the urban areas, and four or more in the rural areas. Interviews were conducted by trained data collectors who could communicate in the local language.

We used Epi-Info version 3.5.3 for data entry. The statistical analysis used Stata version 12. The dependent variable in this study was breastfeeding status, categorized into EBF and non-EBF since birth. The chi-square test or Cochran-Armitage test for categorical variables and Mann-Whitney U test for continuous variables were used to examine the associations between EBF status and other factors. A logistic regression model was used to analyze simultaneous effects of factors on EBF status. Independent variables were entered into logistic regression models if the *p* - values associated with the chi-square test, Cochran-Armitage test and Mann-Whitney U test were less than 0.2. They were infant’s sex and age, mother’s age and level of education, father’s education level and occupation, parity, experience of child death, place of delivery, problems with delivery including Caesarean section, early initiation of breastfeeding and socioeconomic status for both urban and rural areas. Types of delivery assistant were included for rural areas. They were retained in the model if their *p* - values were less than 0.10. The odds ratios with 95% confidence intervals (95% CI) were calculated to assess the adjusted risk of independent variables. Separate analyses were done for the urban and rural areas separately, and a *p* - value less than 0.05 was considered statistically significant.

## Results

A total of 523 mothers were recruited into this study. Five were excluded from the analysis because they did not complete the interviews, and one was excluded because her age was missing. This left 517 cases, 234 urban and 283 rural cases for analysis.

Table 1 shows characteristics of the participating mothers and infants by EBF since birth and residence. In both urban and rural areas, almost half of the infants were male (53.0% in urban and 48.4% in rural). The urban and rural infant’s age distribution indicated that about two-fifth and one-fourth were 0–3 months of age, respectively. Regarding the mother’s age, 67.1% of urban and 76.3% of rural mothers aged < 30 years. Of urban mothers, 50.9% had had any education, whereas 12.0% of rural mothers had some education. For fathers, the percentages were 51.6% for urban and 23.2% for rural. The majority of mothers in both areas were housewives (71.8 and 85.1%). In addition, 93.6% of rural fathers were farmers and 63.7% of urban fathers were self-employed and 26.9% were employees.

**Table 1:** Correlates of exclusive breastfeeding practices in rural and urban Niger: a community-based cross-sectional study

Variable	Urban		*p* - value	Rural		*p* - value
	EBF (*n* = 37) *n* (%)	Non-EBF (*n* = 197) *n* (%)		EBF (*n* = 154) *n* (%)	Non-EBF (*n* = 129) *n* (%)	
Sex of infant						
Male	25 (67.6)	99 (50.3)		79 (51.3)	58 (45.0)	
Female	12 (32.4)	98 (49.7)	0.053	75 (48.7)	71 (55.0)	0.288
Infant’s age						
0-1 m	6 (16.2)	23 (11.7)		6 (3.9)	3 (2.3)	
1-2 m	9 (24.3)	26 (13.2)		19 (12.3)	11 (8.5)	
2-3 m	4 (10.8)	30 (15.2)		19 (12.3)	16 (12.4)	
3-4 m	11 (29.7)	31 (15.7)		26 (16.9)	27 (20.9)	
4-5 m	3 (8.1)	26 (13.2)		40 (26.0)	36 (27.9)	
5-6 m	4 (10.8)	31 (15.7)		25 (16.2)	14 (10.9)	
6 − 7 m	0 (0.0)	30 (15.2)	0.160^a^	19 (12.3)	22 (17.1)	0.484^a^
Mother’s age						
< 30	26 (70.3)	131 (66.5)		110 (71.4)	106 (82.2)	
≥ 30	11 (29.7)	66 (33.5)	0.654	44 (28.6)	23 (17.8)	0.034
Mother’s education						
No education	20 (54.1)	90 (45.7)		141 (91.6)	108 (83.7)	
Any education	14 (37.8)	100 (50.8)		13 (8.4)	21 (16.3)	
Unknown	3 (8.1)	7 (3.6)	0.219	0 (0.0)	0 (0.0)	0.043
Father’s education						
No education	19 (51.4)	85 (43.1)		126 (81.8)	89 (69.0)	
Any education	15 (40.5)	96 (48.7)		26 (16.9)	39 (30.2)	
Unknown	3 (8.1)	16 (8.1)	0.340	2 (1.3)	1 (0.8)	0.008
Mother’s job						
Housewife	24 (64.9)	144 (73.1)		130 (84.4)	110 (85.3)	
Agriculture/Self-employed	7 (18.9)	31 (15.7)		20 (13.0)	19 (14.7)	
Employee	4 (10.8)	13 (6.6)		1 (0.6)	0 (0.0)	
Others	2 (5.4)	9 (4.6)		2 (1.3)	0 (0.0)	
Unknown	0 (0.0)	0 (0.0)	0.730	1 (0.6)	0 (0.0)	0.446
Father’s job						
Famer	1 (2.7)	3 (1.5)		147 (95.5)	117 (90.7)	
Self-employed	26 (70.3)	123 (62.4)		3 (1.9)	9 (7.0)	
Employee	6 (16.2)	57 (28.9)		2 (1.3)	1 (0.8)	
Others	4 (10.8)	14 (7.1)		1 (0.6)	2 (1.6)	
Unknown	0 (0.0)	0 (0.0)	0.395	1 (0.6)	0 (0.0)	0.167
Wealth index						
Lower	16 (43.2)	107 (54.3)		91 (59.1)	49 (38.0)	
Higher	21 (56.8)	90 (45.7)	0.216	63 (40.9)	80 (62.0)	< 0.001
Parity						
1	8 (21.6)	47 (23.9)		13 (8.4)	26 (20.2)	
2–3	19 (51.4)	75 (38.1)		43 (27.9)	38 (29.5)	
4–6	9 (24.3)	56 (28.4)		52 (33.8)	40 (31.0)	
-7	1 (2.7)	19 (9.6)	0.329^b^	46 (29.9)	25 (19.4)	0.004^b^
Child death						
Yes	2 (5.4)	35 (17.8)		67 (43.5)	36 (27.9)	
No	35 (94.6)	162 (82.2)	0.059	87 (56.5)	93 (72.1)	0.007
Antenatal care						
Yes	37 (100.0)	196 (99.5)		138 (89.6)	116 (89.9)	
No	0 (0.0)	1 (0.5)		15 (9.7)	13 (10.1)	
Unknown	0 (0.0)	0 (0.0)	0.664	1 (0.6)	0 (0.0)	0.939
Place of delivery						
Health facilities	34 (91.9)	184 (93.4)		103 (66.9)	103 (79.8)	
Home	3 (8.1)	13 (6.6)		48 (31.2)	25 (19.4)	
Others	0 (0.0)	0 (0.0)		2 (1.3)	1 (0.8)	
Unknown	0 (0.0)	0 (0.0)	0.739	1 (0.6)	0 (0.0)	0.061
Type of birth attendant						
Professional	34 (91.9)	184 (93.4)		108 (70.1)	105 (81.4)	
TBA	0 (0.0)	4 (2.0)		27 (17.5)	8 (6.2)	
No professional	3 (8.1)	9 (4.6)		17 (11.0)	15 (11.6)	
Unknown	0 (0.0)	0 (0.0)	0.468	2 (1.3)	1 (0.8)	0.014
Problems with delivery						
Normal	31 (83.8)	187 (94.9)		151 (98.1)	124 (96.1)	
Caesarean and others	6 (16.2)	10 (5.1)	0.014	3 (1.9)	5 (3.9)	0.330
Postnatal care^c^						
Yes	18 (81.8)	133 (89.9)		111 (86.0)	98 (85.2)	
No	1 (4.5)	9 (6.1)		17 (13.2)	16 (13.9)	
Unknown	3 (13.6)	6 (4.1)	0.855	1 (0.8)	1 (0.9)	0.865
Colostrum						
Yes	37 (100.0)	194 (98.5)		152 (98.7)	125 (96.9)	
No	0 (0.0)	3 (1.5)	0.450	2 (1.3)	4 (3.1)	0.295
Initiation of Breastfeeding						
Within 1 h	25 (67.6)	137 (69.5)		147 (95.5)	109 (84.5)	
After 1 h	12 (32.4)	60 (30.5)		7 (4.5)	19 (14.7)	
Unknown	0 (0.0)	0 (0.0)	0.811	0 (0.0)	1 (0.8)	0.003

Note: EBF was defined as breastfeeding only with receiving breast milk, oral rehydration solution (ORS), vitamins, minerals and medicines since birth

^a^ Mann-Whitney U test ^b^ Cochran-Armitage test: ^c^170 among 234 urban mothers and 244 among 283 rural mothers with infant aged more than 2 months were used for chi-square test

Table 2 shows feeding practices by residence. In both areas, colostrum was almost universally given to newborns (98.7% in urban and 97.9% in rural) and many mothers also started breastfeeding within an hour of giving birth (69.2 and 90.5%). The dietary history since birth indicated that few urban mothers practiced EBF (15.8%), while predominant breastfeeding and complementary feeding were popular (40.2 and 44.0%). The proportion of mothers who fed only breast milk for 24 h preceding this survey was less than half (40.6%). By contrast, more than half of the rural mothers practiced EBF (54.4%), and few were using complementary feeding (13.1%). Three- quarters of rural mothers gave only breast milk in the 24 h prior to the interview (75.6%). The median duration of EBF was 1 week in urban areas and 2 months in rural areas. Among mothers who were not exclusively breastfeeding at the time of this survey, 85.3% in urban and 62.0% in rural areas used prelacteal feeds and 93.4 and 72.7% had stopped EBF within 1 week of giving birth.

**Table 2:** Infant feeding practice by residence

Variable	Urban	Rural
	(*n* = 234)	(*n* = 283)
	*n* (%)	*n* (%)
Initiation of breastfeeding		
Within 1 h	162 (69.2)	256 (90.5)
After 1 h	72 (30.8)	26 (9.2)
Unknown	0 (0.0)	1 (0.4)
Colostrum feeding		
Yes	231 (98.7)	277 (97.9)
No	3 (1.3)	6 (2.1)
Prelacteal feeding		
Yes	168 (71.8)	80 (28.3)
No	65 (27.8)	203 (71.3)
Unknown	1 (0.4)	0 (0.0)
Feeding status in last 24 h		
Only breastfeeding	95 (40.6)	214 (75.6)
Not only breastfeeding	136 (58.1)	69 (24.4)
Unknown	3 (1.3)	0 (0.0)
Feeding status since birth		
EBF	37 (15.8)	154 (54.4)
Predominant breastfeeding	94 (40.2)	92 (32.5)
Complementary feeding	103 (44.0)	37 (13.1)
Duration of EBF		
Within 1 week after birth	183 (78.2)	88 (31.1)
After 1 week of birth	4 (1.7)	12 (4.2)
-1 month	2 (0.9)	9 (3.2)
1-2 months	0 (0.0)	10 (3.5)
2-3 months	1 (0.4)	0 (0.0)
3-4 months	1 (0.4)	2 (0.7)
4-5 months	2 (0.9)	0 (0.0)
5-6 months	0 (0.0)	0 (0.0)
After 6 months of birth	3 (1.3)	0 (0.0)
EBF continue	37 (15.8)	154 (54.4)
Unknown	1 (0.4)	8 (2.8)

Note: EBF was defined as breastfeeding only with receiving breast milk, ORS, vitamins, minerals and medicines since birth

According to the univariate analysis, problems with delivery including Caesarean section and other temporary episodes of mothers during childbirth, such as non-severe asthmatic attack were significantly associated with practice of EBF in urban areas. On the other hand, in the rural areas, lower educational level for parents, older mother’s age, higher parity, experience of child death, lower socioeconomic status, the assistance of a traditional birth attendant (TBA) and early initiation of breastfeeding were significantly associated with a higher proportion of EBF practice (Table 1).

### Multivariate analysis

In urban areas, mothers of infant 3 months old or younger were significantly more likely to practice EBF than those with infants older than 4 months (AOR 2.78; 95% CI 1.07, 7.21). Problems with delivery including Caesarean section were also related to EBF positively (AOR 3.60; 95% CI 1.17, 11.01) (Table 3). In rural areas, mothers who initiated breastfeeding within 1 h after delivery were more likely to breastfeed their children exclusively (AOR 4.04; 95% CI 1.50, 10.83), as were those whose delivery had been assisted by TBA rather than a health professional (AOR 3.49; 95% CI 1.37, 8.89). Mothers with lower socioeconomic status of the household were more likely to breastfeed exclusively (AOR 1.89; 95% CI 1.12, 3.18) (Table 3).

**Table 3:** Multiple logistic regression of determinants of exclusive breastfeeding

1) Urban area		2) Rural area	
Variables		Variables	
Adjusted OR (95%CI)		Adjusted OR (95%CI)	
Age group (month)		Mother’s age group (years)	
≦ 3 m	2.78 (1.07, 7.21)*	≥ 30	1.71 (0.90, 3.24)
≧ 4 m	1	< 30	1
Problems with delivery		Child death	
Caesarian and others	3.60 (1.17, 11.01)*	Yes	1.72 (0.98, 3.01)
Normal	1	No	1
		Wealth Index	
		Low	1.89 (1.12, 3.18)*
		High	1
		Initiation of breastfeeding	
		Within one hour	4.04 (1.50, 10.83)*
		> one hour	1
		Type of delivery assistance	
		Traditional birth assistant	3.49 (1.37, 8.89)*
		Others	1.01 (0.45, 2.25)
		Health professional	1

Note: EBF was defined as breastfeeding only with receiving breast milk, ORS, vitamins, minerals and medicines since birth

*p - value < 0.05

## Discussion

This study found that EBF was uncommon in both urban and rural areas in Niger though early initiation of breastfeeding and colostrum feeding were consistent with global trends [25, 26]. The proportion of EBF was far below the 90% level required to reduce mortality among children under 5 years of age, though rural mothers were more likely to breastfeed exclusively than the average for the least developed countries (49%) and the West and Central African region (29%) [2, 23]. This may be thanks to presence of TBA as discussed later. Exclusive breastfeeding had stopped most often within 1 week of giving birth in both urban and rural areas. Many mothers ceased EBF because of prelacteal feeding. A high rate of prelacteal feeding has also been reported in other African countries [8, 9, 27]. The mothers may have thought that giving certain liquids and semi-solid (honey) foods would not mean stopping EBF because these foods were given once only, as part of a custom or as medicine. Many Nigerians who are Muslim, have been reported to hold a ceremony giving their new infants water from Mecca or water treated by a religious leader, as part of a prayer for the infant’s health and intelligence. As part of a local cultural tradition in Niger, infants are often given orange juice and a decoction as prelacteal feeds and this is a common practice among African mothers [8]. Mothers in this study also gave orange juice, decoction and honey to treat infant illnesses. Local culture therefore remains one of the biggest obstacles to EBF for 6 months in Niger, where coordination between local cultures and EBF needs to be established.

In this study, sociodemographic factors associated with EBF included younger infants in urban areas and lower socioeconomic status in rural areas. These findings are consistent with other studies in low and middle income countries [15, 16, 28]. As previous studies showed, rural mothers who practiced early initiation of breastfeeding tended to breastfeed exclusively [16, 29]. Urban mothers who had problems with delivery including Caesarean section and rural mothers whose deliveries had been assisted by TBA were more likely to breastfeed exclusively though these positive effects on EBF are not consistent with other studies [7, 30–35]. Both results may reveal a key point to promote EBF, that there be sufficient communication with a professional at the time of delivery. As for problems with delivery including Caesarean section, some studies reported failure of practicing EBF because of discomfort and delay giving of breast milk by mother-infant separation [16, 30, 34–36]. However, mother-infant separation at hospital after Caesarean section did not occur in the urban areas of this study because of congestion with patients in the limited number of the postnatal rooms [17]. In addition, urban mothers having problems with delivery may become more conscious of the importance of EBF and receive additional information on EBF from health professionals during longer hospitalization. A mother’s contact with health services affects EBF positively because of greater exposure to information about breastfeeding from health professionals [7, 37–39]. However, just getting information through antenatal care is not sufficient for behavioral change [33]. Shirima et al. [32] reported that information given before discharge after normal delivery at a hospital was not related to practice of EBF because of the short duration of the hospital stay and maternal fatigue after normal delivery. Several opportunities for mothers to obtain adequate information about breastfeeding in appropriate situations should be provided to promote EBF as shown in other studies [32, 40, 41]. In the rural areas of our study, at least one TBA in each village in this study had been trained about optimal breastfeeding. They played a role similar to health professionals in urban areas. Information about optimal breastfeeding from TBAs who are familiar to women may improve practice of EBF though this has not been shown in previous studies [7, 37–39]. In rural areas, a TBA has a considerable influence on pregnancy and delivery, greater than health professionals and the pregnant women’s mothers. On the other hand, the influence of TBA has become more limited in urban areas and they have largely been replaced by health professional [42]. This study confirmed that the practice of EBF increased following enough communication about breastfeeding with professionals, whether health professionals for urban mothers or TBA for rural mothers.

This study has some limitations. The assessment of feeding by retrospective self-report was less valid than by observation of feeding for a considerable period, because information on feeding history depended on only the mother’s recall. Idealized breastfeeding practices could have been reported because of social desirability bias since all information was obtained from face-to-face interviews. It could also be possible that some rural mothers made false reports about feeding history to receive aid during a food crisis in 2012.

## Conclusions

In this study, the practice of EBF for infants under 7 months was not common though feeding colostrum and early initiation of breastfeeding were universal. Many mothers stopped EBF early in both urban and rural areas because of prelacteal feeding. Urban mothers with younger infants, had problems with delivery, and rural mothers with low socioeconomic status, early initiation of breastfeeding and delivery assistance by TBA were more likely to practice exclusive breastfeeding. These findings suggest that mothers need adequate information about optimal breastfeeding by professionals, at the time of delivery. Training health professionals including TBA not only on the promoters but also on the barriers of EBF such as prelacteal feeding can be a practical action the country can take forward. Their supportive individual counseling or group education for mothers and their families about breastfeeding, including the reassurance that there is no need to give the baby any other drink or food, could help to improve levels of EBF in Niger.

## Additional file

Additional file 1: Questionnaire on correlates of exclusive breastfeeding practices in Niger. (XLSX 20 kb)

## Data Availability

The data that supports the findings of the current study are available from the corresponding author upon reasonable request.

## Abbreviations

AOR: Adjusted odds ratio
CI: Confidence interval
EBF: Exclusive breastfeeding
TBA: Traditional birth attendant

## References

[1] Victora CG, Bahl R, Barros AJ, Franca GV, Horton S, Krasevec J, Murch S, Sankar MJ, Walker N, Rollins NC (2016). Breastfeeding in the 21st century: epidemiology, mechanisms, and lifelong effect. Lancet..

[2] UNICEF. The state of the World's children 2017: Children in Digital World; https://www.unicef.org/publications/files/SOWC_2017_ENG_WEB.pdf. Accessed 26 Oct 2018.

[3] Ford K, Labbok M (1990). Who is breast-feeding? Implications of associated social and biomedical variables for research on the consequences of method of infant feeding. Am J Clin Nutr.

[4] Rasheed S, Frongillo EA, Devine CM, Alam DS, Rasmussen KM (2009). Maternal, infant, and household factors are associated with breast-feeding trajectories during infants’ first 6 months of life in Matlab, Bangladesh. J Nutr.

[5] Duong DV, Binns CW, Lee AH (2004). Breast-feeding initiation and exclusive breast-feeding in rural Vietnam. Public Health Nutr.

[6] Agho KE, Dibley MJ, Odiase JI, Ogbonmwan SM (2011). Determinants of exclusive breastfeeding in Nigeria. BMC Pregnancy Childbirth.

[7] Aidam BA, Perez-Escamilla R, Lartey A, Aidam J (2005). Factors associated with exclusive breastfeeding in Accra, Ghana. Eur J Clin Nutr.

[8] Davies-Adetugbo AA (1997). Sociocultural factors and the promotion of exclusive breastfeeding in rural Yoruba communities of Osun state, Nigeria. Soc Sci Med.

[9] Engebretsen IM, Wamani H, Karamagi C, Semiyaga N, Tumwine J, Tylleskar T (2007). Low adherence to exclusive breastfeeding in eastern Uganda: a community-based cross-sectional study comparing dietary recall since birth with 24-hour recall. BMC Pediatr.

[10] Falceto OG, Giugliani ER, Fernandes CL (2004). Influence of parental mental health on early termination of breast-feeding: a case-control study. J Am Board Fam Pract.

[11] Ghosh R, Mascie-Taylor CG, Rosetta L (2006). Longitudinal study of the frequency and duration of breastfeeding in rural Bangladeshi women. Am J Hum Biol.

[12] Li R, Darling N, Maurice E, Barker L, Grummer-Strawn LM (2005). Breastfeeding rates in the United States by characteristics of the child, mother, or family: the 2002 National Immunization Survey. Pediatrics..

[13] Milligan RA, Pugh LC, Bronner YL, Spatz DL, Brown LP (2000). Breastfeeding duration among low income women. J Midwifery Womens Health.

[14] Patel A, Badhoniya N, Khadse S, Senarath U, Agho KE, Dibley MJ (2010). South Asia infant feeding research N. infant and young child feeding indicators and determinants of poor feeding practices in India: secondary data analysis of National Family Health Survey 2005-06. Food Nutr Bull.

[15] Shirima R, Greiner T, Kylberg E, Gebre-Medhin M (2001). Exclusive breast-feeding is rarely practised in rural and urban Morogoro, Tanzania. Public Health Nutr.

[16] Rollins NC, Bhandari N, Hajeebhoy N, Horton S, Lutter CK, Martines JC, Piwoz EG, Richter LM, Victora CG (2016). Why invest, and what it will take to improve breastfeeding practices?. Lancet..

[17] Ministere de la sante Publique. Annuaire des statistiques sanitaires du niger; http://www.stat-niger.org/statistique/file/Annuaires_Statistiques/snis/Ann_stat_2012_Niger.pdf. Accessed 26 Oct 2018.

[18] International vaccine access Center at the Johns Hopkins Bloomberg School of Public Health. The 2016 pneumonia and diarrhea progress report: reaching goals through action and innovation; http://www.ipa-world.org/uploadedbyfck/IVAC-2016-Pneumonia-Diarrhea-Progress-Report.pdf. Accessed 26 Oct 2018.

[19] UNICEF. Under-five mortality; https://data.unicef.org/topic/child-survival/under-five-mortality/. Accessed 11 Feb 2019.

[20] République Du Niger MDLSP-N. Document de stratégie nationale de survie de l'enfant; http://www.aho.afro.who.int/profiles_information/images/f/f1/R347MOHNiger_2008_Strategie_nationale_Survie_de_lEnfant.pdf. Accessed 23 July 2019.

[21] IBFAN. State of the code by country; http://www.ibfan.org/art/298-11.pdf. Accessed 26 Feb 2018.

[22] Le Gouvernement de la République du NIGER, UNICEF. Plan d'action du programme de pays 2014-2018; file:///C:/Users/kaneko/Documents/EBF (MPH).Data/PDF/Niger_CPAP_2014-2018.pdf. Accessed 22 Feb 2019.

[23] Institut National de la Statistique (INS) MdF. Enquête Démographique et de Santé et à Indicateurs Multiples [The Demographic and Health Surveys] https://dhsprogram.com/what-we-do/survey/survey-display-407.cfm. Accessed 10 Oct 2018.

[24] WHO. Indicators for assessing infant and young child feeding practices-part1 definitions; http://www.who.int/maternal_child_adolescent/documents/9789241596664/en/. Accessed 26 Oct 2018.

[25] Cai X, Wardlaw T, Brown DW (2012). Global trends in exclusive breastfeeding. Int Breastfeed J.

[26] UNICEF. Breastfeeding; Available at : <http://www.unicef.org/nutrition/index_24824.html>. Accessed 26 Oct 2012.

[27] Diagne-Guye NR, Diack-Mbaye A, Dramé M, Diagne I, Fall AL, Camara B (2011). Connaissances et pratiques de mères sénégalaises vivant en milieu rural ou suburbain sur l'alimentation de leurs enfants, de la naissance à l'âge de six mois [Knowledge and practices of Senegalese mothers living in rural or suburban area on children feeding from birth to six months of age]. J Pédiatr Puéric.

[28] Perez-Escamilla R, Lutter C, Segall AM, Rivera A, Trevino-Siller S, Sanghvi T (1995). Exclusive breast-feeding duration is associated with attitudinal, socioeconomic and biocultural determinants in three Latin American countries. J Nutr.

[29] Balogun OO, Dagvadorj A, Anigo KM, Ota E, Sasaki S (2015). Factors influencing breastfeeding exclusivity during the first 6 months of life in developing countries: a quantitative and qualitative systematic review. Matern Child Nutr.

[30] Prior E, Santhakumaran S, Gale C, Philipps LH, Modi N, Hyde MJ (2012). Breastfeeding after cesarean delivery: a systematic review and meta-analysis of world literature. Am J Clin Nutr.

[31] Issaka AI, Agho KE, Page AN, Burns PL, Stevens GJ, Dibley MJ (2015). Factors associated with early introduction of formula and/or solid, semi-solid or soft foods in seven francophone west African countries. Nutrients..

[32] Shirima R, Gebre-Medhin M, Greiner T (2001). Information and socioeconomic factors associated with early breastfeeding practices in rural and urban Morogoro, Tanzania. Acta Paediatr.

[33] Horii N, Allman J, Martin-Prevel Y, Waltisperger D (2017). Determinants of early initiation of breastfeeding in rural Niger: cross-sectional study of community based child healthcare promotion. Int Breastfeed J.

[34] Shifraw T, Worku A, Berhane Y (2015). Factors associated exclusive breastfeeding practices of urban women in Addis Ababa public health centers, Ethiopia: a cross sectional study. Int Breastfeed J.

[35] Seid AM, Yesuf ME, Koye DN (2013). Prevalence of exclusive Breastfeeding practices and associated factors among mothers in Bahir Dar City, Northwest Ethiopia: a community based cross-sectional study. Int Breastfeed J.

[36] Esteves TM, Daumas RP, Oliveira MI, Andrade CA, Leite IC (2014). Factors associated to breastfeeding in the first hour of life: systematic review. Rev Saude Publica.

[37] Dibley MJ, Roy SK, Senarath U, Patel A, Tiwari K, Agho KE, Mihrshahi S (2010). Across-country comparisons of selected infant and young child feeding indicators and associated factors in four south asian countries. Food Nutr Bull.

[38] Ogunlesi TA (2010). Maternal socio-demographic factors influencing the initiation and exclusivity of breastfeeding in a Nigerian semi-urban setting. Matern Child Health J.

[39] Dennis CL (2002). Breastfeeding initiation and duration: a 1990-2000 literature review. J Obstet Gynecol Neonatal Nurs.

[40] Ekambaram M, Vishnu Bhat B, Ahamed MAP (2010). Knowledge, attitiude and practice of breastfeeding among postnatal mothers. Curr Pediatr Res.

[41] Ludvigsson JF (2003). Breastfeeding in Bolivia - information and attitudes. BMC Pediatr.

[42] Brighton A, D’Arcy R, Kirtley S, Kennedy S (2013). Perceptions of prenatal and obstetric care in sub-Saharan Africa. Int J Gynaecol Obstet.

